# Single cell resolution *in vivo* imaging of DNA damage following PARP inhibition

**DOI:** 10.1038/srep10129

**Published:** 2015-05-18

**Authors:** Katherine S. Yang, Rainer H. Kohler, Matthieu Landon, Randy Giedt, Ralph Weissleder

**Affiliations:** 1 Center for Systems Biology, Massachusetts General Hospital, 185 Cambridge St, CPZN 5206, Boston, MA 02114; 2 Department of Systems Biology, Harvard Medical School, 200 Longwood Ave, Boston, MA 02115.

## Abstract

Targeting DNA repair pathways is a powerful strategy to treat cancers. To gauge efficacy *in vivo*, typical response markers include late stage effects such as tumor shrinkage, progression free survival, or invasive repeat biopsies. These approaches are often difficult to answer critical questions such as how a given drug affects single cell populations as a function of dose and time, distance from microvessels or how drug concentration (pharmacokinetics) correlates with DNA damage (pharmacodynamics). Here, we established a single-cell *in vivo* pharmacodynamic imaging read-out based on a truncated 53BP1 double-strand break reporter to determine whether or not poly(ADP-ribose) polymerase (PARP) inhibitor treatment leads to accumulation of DNA damage. Using this reporter, we show that not all PARP inhibitor treated tumors incur an increase in DNA damage. The method provides a framework for single cell analysis of cancer therapeutics *in vivo*.

Poly(ADP-ribose) polymerase 1 (PARP1) is a nuclear enzyme that utilizes nicotinamide-adenine-dinucleotide (NAD^+^) to catalyze the addition of poly(ADP-ribose) (PAR) moieties onto target proteins^1^,^2^. These bulky, negatively charged PAR polymers affect numerous cellular processes, most notably by changing the localization and activity of signaling proteins^3^,^4^,^5^. PARP1 promotes DNA repair by undergoing a conformational change in response to single-strand and double-strand DNA breaks^6^,^7^,^8^. PARylation by PARP1 then leads to the recruitment of early DNA damage response factors, such as XRCC1, among many others^9^,^10^,^11^,^12^,^13^,^14^,^15^. Small molecule PARP inhibitors were originally developed for combined use with DNA damaging agents upon discovery that PARP1 is activated following either ionizing radiation or treatment with DNA-methylating agents^4^,^8^,^16^. In addition, the synthetic lethal relationship between PARP1 and BRCA has been exploited in several clinical trials^4^.

Most research on the mechanism of action and failure of PARP inhibitors has been limited to cell culture or traditional pharmacodynamic (PD) assays. These PD assays include measurements on tissue samples and traditional pharmacodynamic measurements of tumor volume following repeated inhibitor treatment in mice bearing BRCA wild-type or BRCA mutant tumors^17^,^18^,^19^. While these approaches are important towards developing more efficient PARP inhibitors, they often do not allow one to study effects at the single cell level *in vivo*. For example, there are a number of questions related to the mechanism of *in vivo* action (PD) such as: is the mechanism of action the same for every cell within the tumor or is there heterogeneity? Are there differences in response mechanisms within tumor classes (i.e. different models of ovarian cancer)? Do host cells impact efficacy? Which combination therapies work synergistically (mechanistically within cells and between tumor and host cells)?

A critical aspect in understanding drug action *in vivo* are robust response markers which yield sufficiently high signal-to-noise to be imaged in real time and which can be imaged in semiautomated fashion. Here we created a double-strand break (DSB) DNA damage reporter based on truncated 53BP1^20^ and using this approach, we measured the single-cell pharmacodynamics of olaparib in different xenografts of human tumor-derived ovarian, breast, and Ewing’s sarcoma cancers^21^ (Fig. 1a). Our results surprisingly show that i) DNA damage can be measured *in vivo*, ii) that, in the case of olaparib, *in vitro* measurements do not predict *in vivo* effects and iii) that there is no clear relationship between BRCA1 status and sensitivity to the PARP inhibitor olaparib, at least in the studied xenografts of ovarian, breast and Ewing’s sarcoma tumors. The studies described here provide a framework for testing the single-cell pharmacodynamics of other DNA damaging agents.

## Results

### Development of a single-cell pharmacodynamic DNA damage reporter

**Figure 1:**
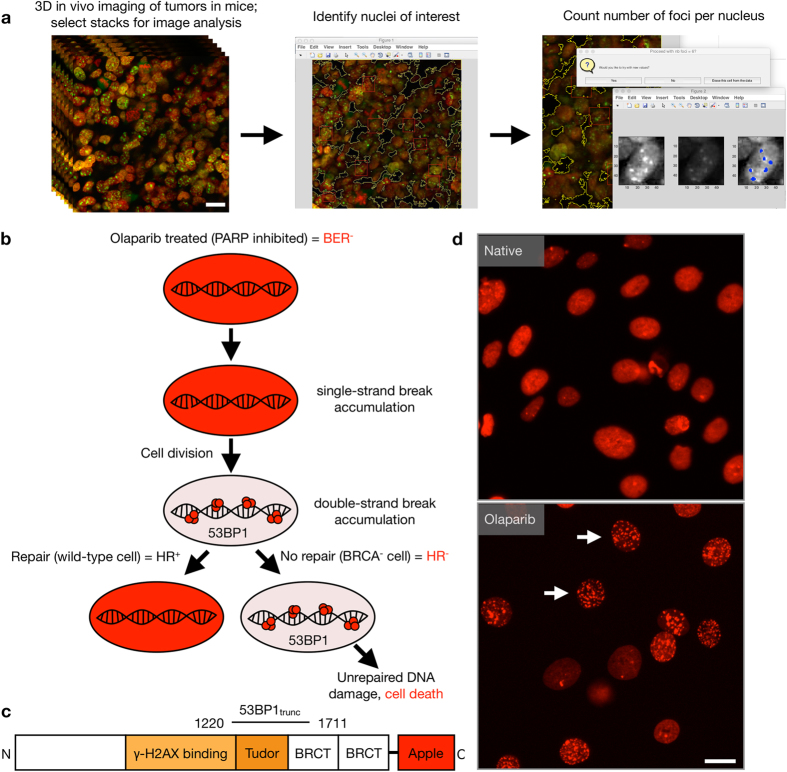
Single-cell pharmacodynamic imaging analysis of olaparib efficacy. (**a**) Image stacks from 53BP1_trunc_-Apple/H2B-GFP *in vivo* imaging experiments were analyzed using MATLAB to determine the number of foci on a per nucleus basis. Individual non-overlapping stacks were selected from a region of tumor imaging for analysis (a, left). Nuclei of interest were identified manually (a, center, red squares) and then the number of foci per nucleus were counted (a, right). The resulting number of foci per nucleus were plotted on a single-cell basis to determine if olaparib treatment caused an increase in the number of 53BP1 foci. Scale bar = 20 μM. (**b**) Synthetic lethality in the context of PARPi and BRCA mutation. Treatment with PARP inhibitors leads to a block in the base excision repair pathway (BER-), which results in accumulation of single-strand DNA breaks. During cell division, these breaks accumulate as double-strand DNA breaks. One of the proteins that accumulates at sites of double-strand breaks is 53BP1 (red foci). In BRCA wild-type cells (homologous recombination positive, HR+), the double-strand breaks are repaired and the cell remains viable. However, in BRCA mutant cells (BRCA-), homologous recombination is blocked (HR-), which leads to sustained 53BP1 accumulation, unrepaired DNA damage, and eventual cell death. (**c**) Schematic of the 53BP1_trunc_-Apple (amino acids 1220–1711)^20^ reporter for *in vivo* single-cell pharmacodynamics. (**d**) Example of PARPi (olaparib) induced increase in 53BP1 foci in an ovarian cancer cell line (OVCAR429) after 24 hrs of 100 μM olaparib. Scale bar = 20 μM.

To develop an *in vivo* single-cell pharmacodynamic assay to measure DSBs following olaparib treatment, we chose 53BP1 as a DSB reporter, which has previously been used to measure DNA damage in live cells^20^,^22^,^23^,^24^,^25^,^26^(Fig. 1b). Specifically, we fused a truncated portion of 53BP1 (amino acids 1220-1711) to Apple fluorescent protein (53BP1_trunc_-Apple)^20^ (Fig. 1c). Apple fluorescent protein was used for *in vivo* imaging due to its increased brightness over mCherry, which is critical for successful imaging in live tissue. The truncated version of 53BP1 retains its ability to bind to sites of DSBs, but lacks the known functional domains of 53BP1^20^ (Fig. 1d). Moreover, we show that 53BP1_trunc_-Apple localizes to sites of double-strand breaks with an antibody targeting the canonical marker of double-strand breaks, γH2A.X (Supplementary Fig. S1).

### Relationship between BRCA1 status and PARP expression

We chose a panel of breast and ovarian cancer cell lines with either BRCA1 wild-type or mutant status, as well as several Ewing’s sarcoma cell lines that are BRCA1 wild-type, but had been shown to be sensitive to PARP inhibitors^21^ (Supplementary Table S1). Among these cell lines, we were first interested in whether or not BRCA1 status correlated with PARP1 expression and if PARP1 expression could therefore predict olaparib sensitivity. Western blot analysis revealed no obvious correlation between BRCA1 status and PARP expression (Supplementary Fig. S2). Some BRCA mutant cell lines had very low PARP1 expression (HCC1937), while others had high PARP1 expression (MDA-MB-436). Similarly, the wild-type cell lines ranged from low expressing (OVCAR429) to high expressing (A2780).

### BRCA1 mutant cell lines are generally more sensitive to olaparib *in vitro*

We next compared cell viability following olaparib treatment to confirm the correlation between BRCA1 status and PARP inhibitor sensitivity. Cell lines were plated at low density and were treated with increasing concentrations of olaparib the following day. After six days treatment, viability was measured using a cell viability assay. As expected, the BRCA1 mutant cell lines generally exhibited increased sensitivity to olaparib compared to the wild-type cell lines (Table 1 and Supplementary Table S1, EC_50_ column; Supplementary Fig. S3), with the exception of the HCC1937 BRCA1^−/−^ cell line, which has a relatively high EC_50_ of 3.7 μM. The Ewing’s sarcoma cell lines exhibited sensitivity comparable to that previously reported (MHH-ES1 is more sensitive than the SK-PN-DW cell line)^21^.

### 53BP1 foci generally increase more in BRCA1 mutant and Ewing’s sarcoma cell lines

**Figure 2:**
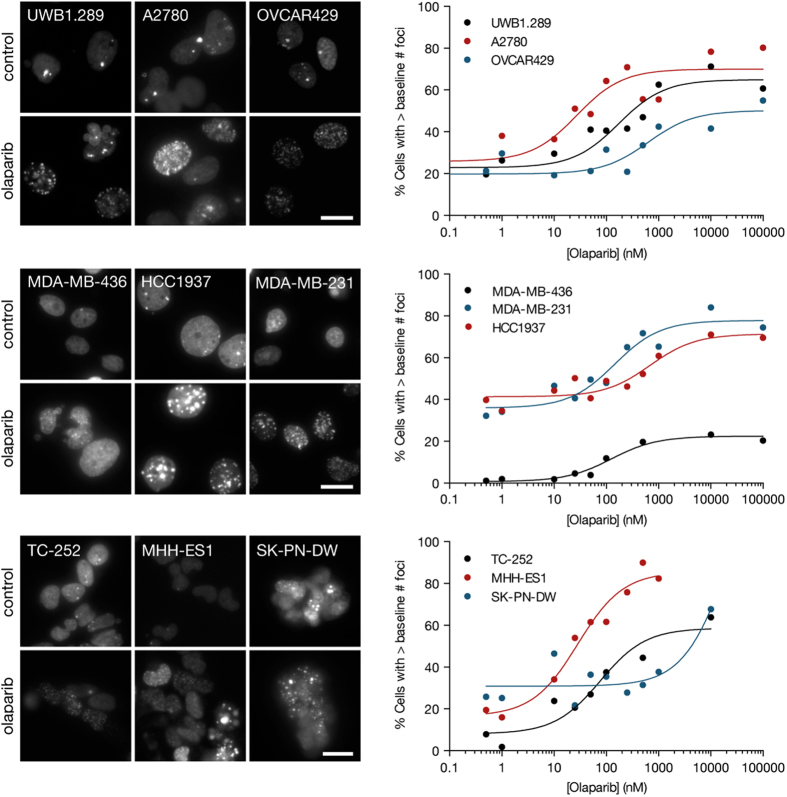
Single-cell pharmacodynamic imaging in cell culture. The indicated cell lines from ovarian, breast, or Ewing’s sarcoma were treated with increasing concentrations of olaparib for 24 hrs, at which point the cells were imaged for 53BP1_trunc_-Apple foci (left). The percentage of cells with greater than the baseline (DMSO control) number of foci was plotted versus olaparib concentration and fit to a sigmoidal dose response curve using Prism (GraphPad) to obtain PD_50_ values (Table 1 and Supplementary Table S1). Data are representative of at least two independent experiments. Scale bar = 20 μM.

**Figure 3:**
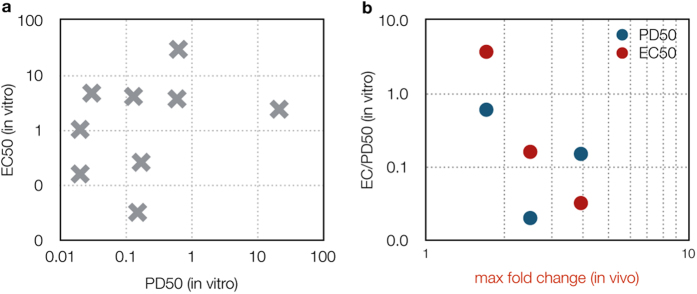
Correlation between pharmacodynamic parameters. (**a**) The half-maximal pharmacodynamic response (PD_50_) from *in vitro* experiments was plotted versus the EC_50_ value from viability assays for the cell lines listed in Supplementary Table S1. (**b**) The maximum fold change in foci formation *in vivo* (x-axis) is plotted versus the half-maximal pharmacodynamic response (PD_50_, blue) or versus the EC_50_ (red) from viability assays.

To determine if DSB accumulation occurs in cell culture following olaparib treatment, all the cell lines listed in Table 1 and Supplementary Table S1 were transduced with lentivirus containing the 53BP1_trunc_-Apple pharmacodynamic reporter. Olaparib induction of DSBs was first measured in culture by single-cell imaging. Each cell line was plated and the next day, increasing concentrations of olaparib were added. Cells were then imaged at 24 hrs (or serially in selected experiments) following olaparib treatment (Fig. 2). For each cell line, the percent increase in 53BP1_trunc_-Apple foci was then determined as a function of time and dose. Drug responses were compared by determining the half-maximal pharmacodynamic response (PD_50_; percent increase in foci/nucleus) as a function of olaparib dose. Interestingly, the PD_50_ values - a measure of DSB effect - do not always correlate with the EC_50_ values, a measure of cell kill (Table 1, Supplementary Table S1, and Fig. 3A). For example, the A2780 ovarian cancer cell line had a high EC_50_ value, but a very low PD_50_ value. This is likely the case because the DSBs that appear after 24 hrs of olaparib treatment are later repaired rather than the cells undergoing cell death (Supplementary Table S1).

In contrast to what the synthetic lethal model would predict for PARP inhibitors, the HCC1937 BRCA1^−/−^ cell line had a high EC_50_ and PD_50_ value. We thus asked if this BRCA1 mutant cell line is either insensitive to olaparib or whether it simply required longer drug incubation to observe an increase in DSBs? To address this question, 53BP1_trunc_-Apple foci formation was imaged and analyzed again at 48 hrs and 6 days after olaparib treatment (this was done for both the HCC1937 and MDA-MB-436 cell lines). Not surprisingly, the PD_50_ values remain the same after 48 hrs of olaparib treatment (Table 1). However, after 6 days olaparib treatment, the PD_50_ values decreased 3-fold for the MDA-MB-436 cells and 7-fold for the HCC1937 cells (Table 1). The increase in DSBs (and resulting decrease in PD_50_) after 6 days of olaparib treatment is consistent with a need for the cells to go through several rounds of cell division to accumulate sufficient numbers of DSBs, particularly at lower concentrations of olaparib. However, for the BRCA1^−/−^ HCC1937 cell line, the increase in DSBs at 6 days (PD_50_) still did not translate to a lower EC_50_ value. It is possible that in this cell line, the additional time required to accumulate DSBs translates into additional time required to observe a decrease in cell viability. Another possibility is that DNA repair pathways in the HCC1937 cell line still function at levels sufficient to repair the damaged DNA. In general, the data in Table 1 and Supplementary Table S1 show that the BRCA mutant cell lines and the expected Ewing’s sarcoma cell lines were sensitive to olaparib.

### Development of an *in vivo* model system to image 53BP1 DNA damage

Ultimately our interest was in measuring DSB accumulation *in vivo*. As an initial validation of the 53BP1_trunc_-Apple reporter *in vivo*, we analyzed the number of foci per nucleus in HT1080 xenografts before and after vehicle or cisplatin (a robust DNA damaging agent) treatment. Supplementary Figure S4 shows that cisplatin treatment resulted in a statistically significant increase in the mean number of foci per nucleus, indicating the 53BP1_trunc_-Apple reporter functions as expected *in vivo*.

**Figure 4:**
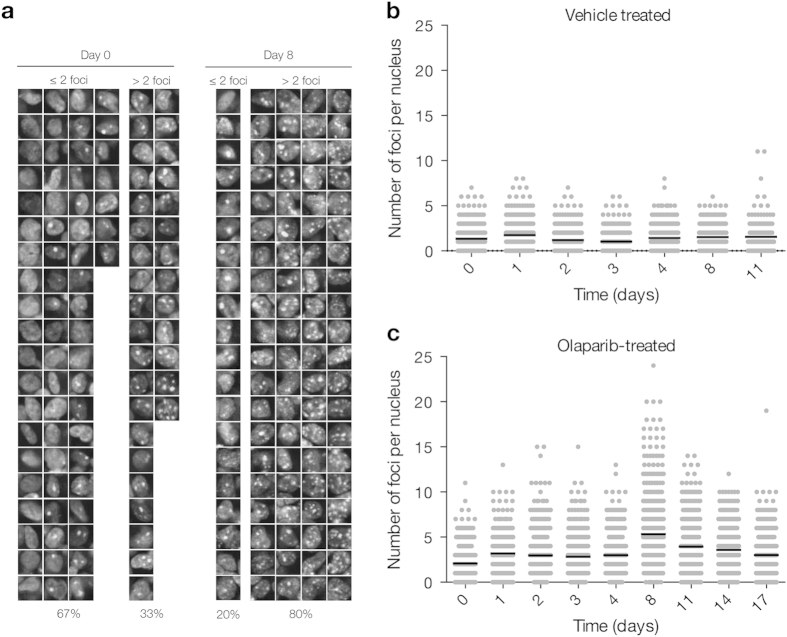
*In vivo* single-cell pharmacodynamic analysis of 53BP1 double-strand break formation following olaparib treatment in Ewing’s sarcoma tumors. (**a**) Representative images of single cells from an MHH-ES1 tumor prior to treatment and after eight days of 100 mg/kg Olaparib. Images are separated into ≤2 foci per nucleus (2 is the average number of foci per nucleus pre-treatment) and >2 foci per nucleus. Percentage values at the bottom represent the percentage of the total number of nuclei that fall into either of these groups. (**b** and **c**) Single cell data for a nu/nu mouse with a Ewing’s sarcoma tumor xenograft (MHH-ES1 cells) that was treated for the duration of imaging with vehicle alone (**b**, 10% DMAC/Solutol in PBS) or with 100 mg/kg Olaparib (**c**). Note for b and c, each point represents the number of foci in a single nucleus, with 200–600 single cells analyzed for each day of imaging. The black line represents the mean number of foci per nucleus for each day of imaging.

To examine the effects of olaparib *in vivo* we established xenograft tumors of cells stably expressing the 53BP1_trunc_-Apple reporter and imaged these tumors at high spatial resolution to resolve the nuclear phenotype of DSB. We focused on three representative models in which serial measurements were carried out at different doses (50 or 100 mg/kg olaparib): MDA-MB-436 (breast cancer), HCC1937 (breast cancer), and MHH-ES1 (Ewing’s sarcoma). Tumors were imaged daily for the first week and then every few days thereafter until imaging was no longer feasible. A MATLAB script was utilized for semi-automated analysis of the single cell data (Fig. 1A). Nuclei regions of interest were identified manually and then the number of foci per nucleus was counted for 200–600 single cells from each tumor for each day of imaging (Fig. 4).

### Ewing’s sarcoma MHH-ES1 tumors exhibit more DNA damage following olaparib treatment

**Figure 5:**
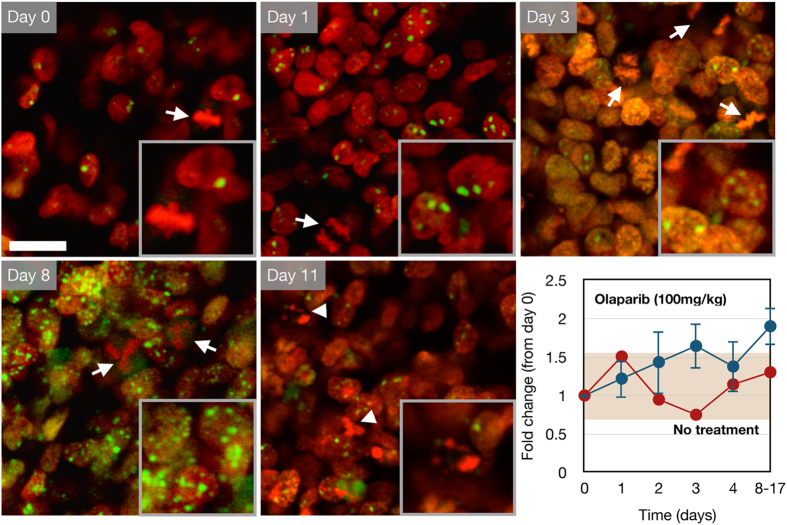
Serial *in vivo* imaging of a nu/nu mouse with a MHH-ES1 Ewing’s sarcoma tumor expressing the 53BP1_trunc_-Apple (green) and H2B-GFP (red) reporter. The mouse was dosed daily with 100 mg/kg olaparib by IP injection (blue curve, n = 3) or vehicle (10% DMAC/Solutol in PBS, n = 1). Days 8–17 indicate pooled data from mice imaged on different days after olaparib treatment. Dividing cells (arrow) and apoptotic cells (arrowhead) were observed using the H2B-GFP (red) reporter. Error bars represent the standard error of the mean. Scale bar = 20 μm (inset magnified 1.7-fold).

**Figure 6:**
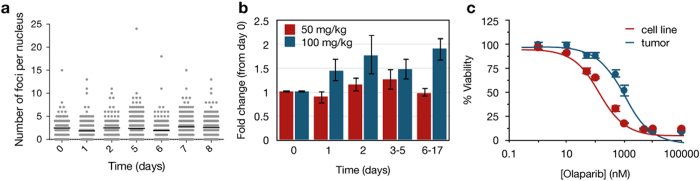
Effect of olaparib concentration on MHH-ES1 Ewing’s sarcoma tumors expressing the 53BP1_trunc_-Apple reporter in nu/nu mice. (**a**) Representative single-cell data showing the number of foci on a per nucleus basis for a mouse treated daily with 50 mg/kg olaparib (IP). Each point indicates the number of foci in a single nucleus, with black lines on each day representing the mean number of foci. (**b**) Comparison of the fold change in the number of foci for the 50 mg/kg (red, n = 2) and 100 mg/kg (blue, n = 3, same data as in Fig. 5 for comparison) olaparib doses (relative to day 0 prior to olaparib treatment). Error bars represent the standard error of the mean. (**c**) Comparison of cell viability following treatment with increasing concentrations of olaparib for 6 days in MHH-ES1 cells grown in culture alone (red) or grown *in vivo* and then dissociated and re-grown in cell culture at the endpoint of olaparib treatment/imaging (blue). Data were fit to a sigmoidal dose-response curve using Prism (GraphPad), with error bars representing the standard error of the mean.

Figure 4 summarizes a set of experiments in the MHH-ES1 model. Examples of the regions of interest for the cells that were imaged each day is shown. Analysis of the fold change in number of foci per nucleus from pre-treatment (day 0) showed a gradual increase with a maximal fold change by day 8 of 100 mg/kg olaparib treatment (Fig. 4 and 5 and Table 1). Importantly, a tumor treated with vehicle (DMSO alone in DMAC/Solutol and PBS) did not show an increase in the average number of foci over time (Fig. 4B and Fig. 5). Interestingly, 50 mg/kg olaparib did not show any effects, but the higher dose of 100 mg/kg did (Fig. 6A and B). These data suggest that there is a dose dependence in the appearance of a pharmacodynamic DSB response. The MHH-ES1 cells in this particular tumor were also transfected with an H2B-GFP reporter that allows visualization of dividing cells, as well as cells that have recently undergone apoptosis^27^. Both of these events are crucial to the efficacy of olaparib treatment. Cell division indicates the ability of cells to accumulate double-strand DNA damage from the olaparib-induced single-strand DNA damage. This phenomenon was observed in the MHH-ES1 cell line following olaparib treatment (Fig. 5). Using the H2B-GFP reporter, we also observed the ultimate apoptotic effects of olaparib (Fig. 5).

At the end point of imaging, MHH-ES1 tumors treated with 100 mg/kg olaparib were isolated and the cells were dissociated and re-grown *in vitro*. Using these cell lines established from tumors treated with olaparib, we repeated the viability assays described earlier. Compared to the cell lines before growth *in vivo*, the tumor cell lines displayed a 6-fold increase in EC_50_ (Fig. 6C). These data suggest that growth and exposure to olaparib *in vivo* fundamentally alters the tumor cells, making them less sensitive to the inhibitor.

### MDA-MB-436 tumors show increased 53BP1 foci after olaparib treatment

**Figure 7:**
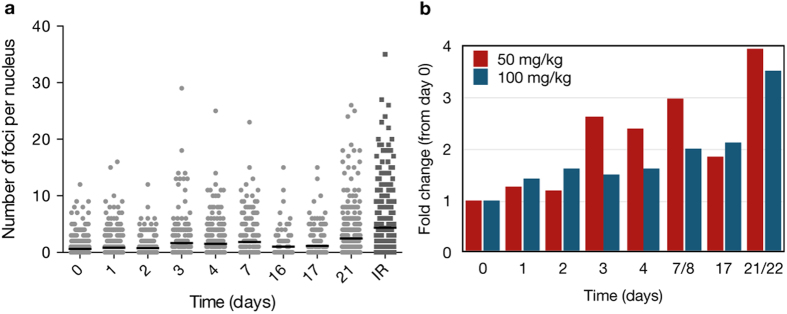
*In vivo* quantification of the number of foci per nucleus in MDA-MB-436 cells expressing the 53BP1_trunc_-Apple reporter in a nu/nu mouse. (**a**) Representative single cell data from a mouse treated with daily IP injections of 50 mg/kg olaparib. Each point represents an individual nucleus *in vivo*, with the y-value equal to the number of foci in that nucleus. 200–600 cells were analyzed each day following olaparib treatment and the black bar represents the average number of foci on each day. (**b**) Comparison of the fold change in the average number of foci per nucleus for daily IP injection of 50 mg/kg (n = 1) versus 100 mg/kg olaparib (n = 1).

Similar analysis as above for Ewing’s sarcoma was also done for MDA-MB-436 BRCA1^−/−^ tumors expressing the imaging reporter (Fig. 7). An increase in the number of foci per nucleus was observed by 21 days following daily olaparib treatment (Fig. 7 and Table 1). Both doses (50 and 100 mg/kg) of olaparib ultimately led to ~4-fold increase in the number of foci per nucleus. Olaparib-treated tumors were also irradiated with 10 gray at the experimental end point to demonstrate that the 53BP1_trunc_-Apple reporter was indeed functional, which was confirmed (Fig. 7A, IR).

### HCC1937 BRCA mutant tumors do not undergo increased 53BP1 foci *in vivo* after olaparib treatment

**Figure 8:**
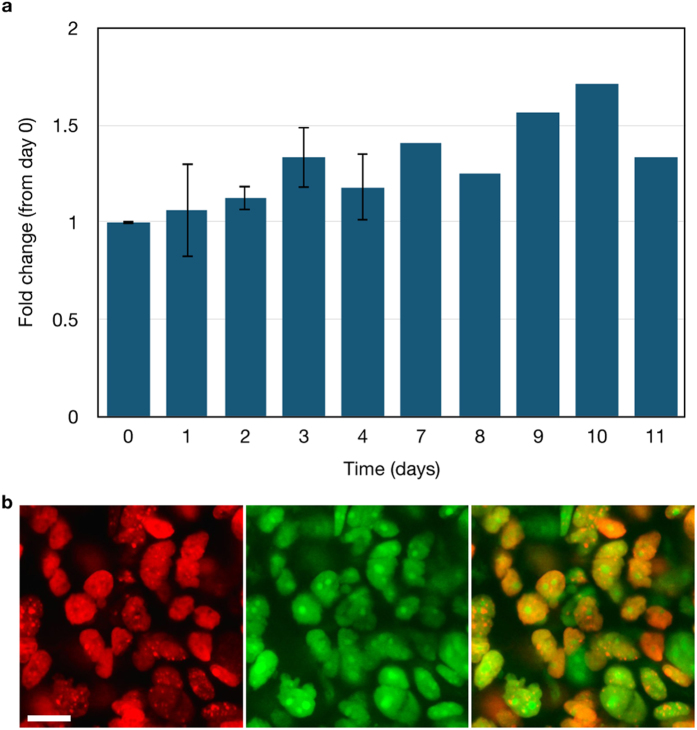
*In vivo* quantification of the fold change in foci formation in HCC1937 53BP1_trunc_-Apple cells grown in nude mice. (**a**) The fold change in the number of foci was determined relative to day 0 of treatment with 100 mg/kg olaparib. Error bars represent the standard error of the mean for n = 2 mice. (**b**) Olaparib-Bodipy FL nuclear uptake in a nu/nu mouse with an HCC1937 tumor. The 53BP1trunc-Apple reporter (red nuclei, left) was imaged 2 hrs after IV administration of olaparib-Bodipy FL (green, center). Images from the 53BP1_trunc_-Apple (red) channel and the olaparib-Bodipy FL (green) channel were overlaid (merge, right) to show that olaparib-Bodipy FL accumulates in all HCC1937 tumor cell nuclei. Scale bar = 20 μm.

Finally, two different HCC1937 BRCA1^−/−^ tumors were studied, each dosed daily with 100 mg/kg olaparib (Fig. 8A). While the maximal fold change was slightly higher in cell culture for this line (~2.5-fold at 6 days olaparib treatment, data not shown), the single-cell tumor imaging showed essentially no fold change from pre-treatment over the 11 day treatment period. This lack of change is also consistent with the viability data for this cell line, which showed relatively poor sensitivity to olaparib (Table 1). The lack of a pharmacodynamic response from the HCC1937 cell line *in vivo* is not due to poor pharmacokinetics of olaparib on the tumor. Using a previously established olaparib-BODIPY FL companion imaging drug^28^ and an HCC1937 53BP1_trunc_-Apple tumor, we observed uptake of the drug in all the tumor cell nuclei (Fig. 8B). This suggests that not all BRCA1 mutant tumors undergo a DNA damage response following olaparib treatment. However, it is possible that the BRCA1 mutant tumor would shrink and respond to olaparib, perhaps through a different and yet unrealized mechanism.

## Discussion

The synthetic lethal relationship between PARP1 and BRCA has been the firm basis of designing recent clinical trials^4^. These trials showed promising results early on in BRCA deficient breast and ovarian cancer with the inhibitor olaparib^29^. Based on these early advancements, the target patient population was expanded to include those that displayed BRCAness, where BRCA1 and BRCA2 are intact, but other HR defects exist. Iniparib showed early promise in a phase 2 trial in triple-negative breast cancer (TNBC) patients that had a BRCAness signature^30^. However, in a phase 3 trial, iniparib failed in the same patient population and at the same time olaparib showed no overall benefit in TNBC patients or overall survival in ovarian cancer patients^31^,^32^,^33^. While PARP inhibitor trials were temporarily halted, more recent studies show that iniparib was not a true PARP inhibitor^34^,^35^. Moreover, olaparib was reevaluated based on BRCA status, with results showing overall survival in patients with defective BRCA protein. Despite the sound scientific rationale^36^,^37^,^38^, extensive *in vitro* assay and preclinical studies^17^,^18^,^19^,^39^,^40^,^41^, the clinical results were mixed^29^,^30^,^31^,^32^,^33^,^42^,^43^,^44^. Furthermore, previous studies using fluorescent olaparib had shown that the drug clearly distributes throughout tumors and has a unique nuclear phenotype of accumulation *in vivo*^28^. Finally, mathematical modeling had shown that the human doses would clearly result in efficacy in cancer cells^28^. So, how can these discrepant results be explained?

In order to gain insight into the pharmacologic actions of PARP inhibitors, we have created tool sets to measure pharmacokinetics^28^ and now also pharmacodynamics at the single cell level *in vivo* and in real-time. The latter was accomplished by creating a sufficiently bright fluorescent 53BP1 reporter for confocal/multiphoton *in vivo* imaging. This reporter shows a remarkable phenotype (Fig. 1D) and allows one to derive quantitative measures of drug efficacy (EC_50_: concentration at which half of the cells die, PD_50_: concentration at which half of the cells show DNA damage *in vitro*, *in vivo*: maximum fold increase in DNA damage relative to pre-treatment). Our data in cell culture shows that the 53BP1_trunc_-Apple reporter generally agrees with the viability data for each cell line, with the major exception being the HCC1937 cell line. This line is BRCA1 mutant and has a low PD_50_ value after 6 days of olaparib treatment, indicating a strong increase in DSBs, but a high EC_50_ value over the same time frame, which indicates the cells do not die readily in response to olaparib. Perhaps this is owing to the relatively low expression of PARP1 in these cells compared to the other more responsive cell lines.

*In vivo* we show with three different tumor models, two of which are BRCA1 mutant, that there is a difference in the number of DSBs observed depending on the daily dose of olaparib. For one of the cell lines (MHH-ES1), a lower dose of olaparib (50 mg/kg) did not increase the number of DSBs. However, with a dose of 100 mg/kg a significant number of DSBs were observed. Surprisingly, the MDA-MB-436 cell line showed the greatest increase in the number of 53BP1_trunc_-Apple foci, even at the 50 mg/kg olaparib dose. This is in contrast to the cell culture data, which suggested that while the MDA-MB-436 cell line displayed high sensitivity to olaparib in a viability assay, there was only a moderate change in 53BP1_trunc_-Apple foci. Similarly, the HCC1937 BRCA1 mutant cell line showed little change in the number of foci *in vivo*, even at the higher 100 mg/kg daily dose of olaparib.

With certain caveats, the experiments of this study suggest two main conclusions: i) often there is no clear relationship between BRCA1 status and sensitivity to olaparib and ii) *in vitro* cell culture data do not predict *in vivo* efficacy for the PARP inhibitor studied here. While the MDA-MB-436 cell line is sensitive *in vivo*, the HCC1937 cell line is not, despite both lines being BRCA1^−/−^. This suggests that if HCC1937 tumors still decrease in size, it is likely independent of the synthetic lethal concept. PARP1 has a plethora of other functions in the cell ranging from promotion of transcription to chromatin modification to changes in signaling pathways. Modification of any of these pathways/proteins through PARP1 inhibition could in theory affect tumor growth. Recent studies demonstrate that olaparib induces a G2 phase arrest-like state, resulting in upregulation of p53 and p21^45^,^46^,^47^. Furthermore, these studies suggest that olaparib is most effective in cell lines with mutated p53. Here, the cell lines studied *in vivo* all have mutant p53 status^48^ (and canSAR database). Interestingly, the HCC1937 cell line has strong basal expression of p21, while the MDA-MB-436 and MHH-ES1 cell lines do not (Supplementary Fig. S5). Thus, it is possible that the HCC1937 cell line arrests during the cell cycle and does not proliferate at the same rate as the MDA-MB-436 and MHH-ES1 cell lines *in vivo*. Replication is essential to PARP inhibitor function in the context of synthetic lethality (Fig. 1B), suggesting that cell lines that may proliferate more slowly (such as HCC1937) may be less sensitive to olaparib. We also show that the Ewing’s sarcoma tumor is sensitive to olaparib *in vivo*, consistent with previous cell culture data^21^. These data also highlight the importance of *in vivo* measurements of single-cell pharmacodynamics. The cell culture data would predict that all the cell lines we tested *in vivo* would show an increase in DSBs following olaparib treatment, which is clearly not the case. We believe that the methodology we have established here to measure DSBs will be applicable not only to PARP inhibitors, but to other agents that also induce DSBs. Similar types of reporters could also be established for other cell processes so that one can gain a more complete view of drug action *in vivo*.

## Materials and Methods

### Cell lines and growth conditions

The UWB1.289, MDA-MB-436, MDA-MB-231, and SK-PN-DW cell lines were purchased from the American Type Culture Collection (ATCC). A2780 and OVCAR429 cell lines were kind gifts from Dr. Michael Birrer (Massachusetts General Hospital, Boston, MA). The HCC1937 cell line was a kind gift from Prof. Timothy Mitchison (Department of Systems Biology, Harvard Medical School, Boston, MA). The TC-252 cell line was a kind gift from Dr. Francis Hornicek (Massachusetts General Hospital, Boston, MA). MHH-ES1 cells were purchased from CLS Cell Lines Service (order # 300136).

MDA-MB-436, MDA-MB-231, A2780, OVCAR429, MHH-ES1, and HCC1937 cells were maintained in RPMI supplemented with 10% fetal bovine serum, 100 IU penicillin, 100 μg/ml streptomycin, and L-glutamine. UWB1.289 cells were maintained in 50% RPMI, 50% MEGM (consisting of MEBM basal medium and SingleQuot aliquots, excluding gentamycin-amphotericin B; Lonza CC-3150), 3% fetal bovine serum, 100 IU penicillin, 100 μg/ml streptomycin, and L-glutamine. SK-PN-DW, TC-252, and HT1080 cells were maintained in DMEM supplemented with 10% fetal bovine serum, 100 IU penicillin, 100 μg/ml streptomycin, and L-glutamine. Cells expressing 53BP1_trunc_-Apple were maintained in 3 μg/ml puromycin and cells co-expressing H2B-GFP were also maintained in 200 μg/ml G418.

### Constructs

Truncated 53BP1 (amino acids 1220–1711^20^) was PCR amplified from 53BP1-YFP (kind gift from Prof. Galit Lahav, Harvard Medical School, Boston, MA). The PCR product was cloned into the pLVX lentiviral vector (632562, Clontech) containing a C-terminal Apple fluorescent protein (27698, Addgene), using In-Fusion cloning (639645, Clontech) and the BsrGI and XbaI restriction enzyme sites on the pLVX vector. The 53BP1_trunc_-Apple insert was sequenced in its entirety.

The H2B-GFP reporter was created by replacing Apple fluorescent protein in pTag-H2B-Apple^49^ (FP176, Evrogen) with pAcGFP1 (632492, Clontech). pTag-H2B-Apple was digested using the AgeI and NotI restriction enzymes, while pAcGFP1-HyG-C1 was digested using the AgeI and BspOMI restriction enzymes. pAcGFP1 was then ligated into the pTag-H2B vector and the resulting insert was sequenced in its entirety.

### Transfection and lentiviral infection

Lentiviral particles of the 53BP1_trunc_-Apple reporter were produced in Lenti-X 293T cells (632180, Clontech) following the manufacturers’ instructions. Briefly, 2 × 10^6^ Lenti-X 293T cells were plated in a 10 cm dish. The following day, cells were transfected with the pLVX 53BP1_trunc_-Apple reporter using Xfect (631317, Clontech). After 6 hrs, the media was changed to remove transfection complexes. 48 hrs after transfection the media was collected and filtered through 0.45 μm cellulose acetate. Viral particles were then concentrated using Lenti-X concentrator (631231, Clontech). Cells were plated at 50,000 cells per 12-well dish and the following day were transduced with 53BP1_trunc_-Apple lentiviral particles in media containing 4–10 μg/ml polybrene (depending on the cell line). Media was exchanged after 24 hrs and cells were split into media containing 3 μg/ml puromycin for selection after 48 hrs. Reporter expression was confirmed by fluorescence microscopy and cells with uneven expression were sorted by flow cytometry.

For co-expression of H2B-GFP, cells were plated 24 hrs before transfection and the following day, the pTag H2B-GFP plasmid was transfected into cells using Lipofectamine 2000 (11668-027, Life Technologies). Transfected cells were split into media containing 500–1000 μg/ml G418. Cells positive for both 53BP1_trunc_-Apple and H2B-GFP were obtained by FACS or by isolating individual colonies after selection.

### Western blot

Cells were grown to confluence, washed twice with ice-cold PBS and then lysed in radioimmunoprecipitation buffer (RIPA, 9806S, Cell Signaling Technology) containing HALT protease inhibitor cocktail (87786, Pierce). Lysates were transferred to microfuge tubes, passed through a 23 g syringe, and incubated five minutes on ice. Lysates were sonicated for 40 s and then centrifuged at 14,000 × g for 15 min at 4 °C to remove cellular debris. Total protein was measured using the BCA assay (23227, Pierce) and equal protein was loaded on a 4–12% NuPAGE Bis-Tris gel (NP0322BOX, Life Technologies). Protein was then transferred to nitrocellulose and stained for total protein (for normalization) using the reversible protein stain kit for nitrocellulose membranes (24580, Pierce). Following staining and documentation, the stain was removed and the blot was blocked in SuperBlock T20 (TBS) (37536, Pierce), followed by brief washing in TBS containing 0.1% Tween-20 (TBST). Blots were incubated overnight at 4 °C in PARP1 (9532, Cell Signaling Technology), p53 (2527, Cell Signaling Technology), or p21 (2947, Cell Signaling Technology) primary antibody diluted 1:1000 in 10% SuperBlock/TBST. Blots were washed three times, 5 min each, followed by a one hour incubation in HRP-conjugated secondary antibody. Following secondary incubation, cells were again washed three times, 5 min each in TBST followed by detection using SuperSignal West Pico chemiluminescent substrate (34077, Pierce). PARP1 expression was quantified by densitometry using ImageJ (NIH) and normalized using the total protein stain and densitometry of ~80 kDa band in each lane. PARP1 expression was quantified from two independent experiments, with error calculated as standard error of the mean.

### PrestoBlue viability assay

Cells were plated at 1000 to 3000 cells per well (depending on the cell line) 24 hrs prior to addition of olaparib (S1060, Selleckchem). Increasing concentrations of olaparib (0 to 100 μM) were added to each well for six day incubation. Cell viability was measured on the sixth day using the PrestoBlue cell viability reagent (A13261, Life Technologies) and a Tecan Safire2 plate reader in fluorescence mode (Männedorf, Switzerland). Data were plotted as percent viability versus olaparib concentration and the resulting curve was fit to a sigmoidal dose response equation using GraphPad (Prism). Error was calculated as standard error of the mean between triplicate measurements. Data are representative of at least two independent experiments.

### Olaparib 53BP1_trunc_-Apple live cell imaging

Cells were plated at 2000 to 10,000 cells per well (depending on the number of days of olaparib treatment) in 96-well plates for microscopy (89626, iBidi). After 24 hrs, increasing concentrations of olaparib (0–100 μM) were added to the cells. Imaging was done on a DeltaVision (Applied Precision/GE Healthcare) microscope equipped with a 40X objective and a humidified environmental chamber 24 hrs after addition of olaparib (as well as 48 hrs and 6 days for select cell lines).

For colocalization with γH2A.X, cells were fixed with 4% paraformaldehyde after treatment for 24 hrs with 100 μM olaparib or 1 μM etoposide or 1 hr with 10 μM cisplatin (followed by 24 hr recovery). Cells were permeabilized using 100% MeOH, washed with PBST (PBS plus 0.1% Tween-20), blocked with odyssey blocking buffer (Li-COR), and incubated overnight at 4 °C in primary γH2A.X (phospho S139, abcam, ab26350) antibody diluted 1:100 in odyssey blocking buffer. Cells were washed with PBST and incubated 1 hr at room temperature in AlexaFluor 647 goat anti-mouse secondary antibody (1:100, Life Technologies). Cells were then imaged using a DeltaVision microscope equipped with a 40X objective.

### *In vivo* single-cell pharmacodynamic imaging

All animal experiments were carried out in accordance with guidelines from the Institutional Subcommittee on Research Animal Care. Female nude mice (8–10 weeks old, Cox7, Massachusetts General Hospital) were implanted in the dorsal skinfold/subcutaneously with 2–4 × 10^6^ cells that were suspended 1:1 in Hank’s balanced salt solution (HBSS) and Matrigel (356237, BD Biosciences). Tumors were allowed to grow (2 weeks to 2 months depending on the cell type) until they became vascularized and reached 1–2 mm in size. At this point, mice were surgically implanted with a dorsal skin fold window chamber. For the HT1080 experiments, window chambers and cells were implanted at the same time (without matrigel). Prior to olaparib treatment, mice were anesthetized with 2% isoflurane in 2 L/minute oxygen on a heated microscope stage. Day 0 images were collected from various regions of the tumor on an FluoView FV1000 confocal microscope (Olympus) equipped with a 20X water immersion objective. Mice were then treated daily by IP injection with 50 mg/kg olaparib (80 mg/ml stock in DMSO diluted in PBS with 10% DMAC/Solutol) or on a cycle of 5 days dosing, 2 days rest with 100 mg/kg olaparib. Initially, image stacks were collected daily and then every few days during olaparib treatment. For the HT1080 experiments, Cisplatin was injected as a single IV dose of 9 mg cisplatin per kilogram (in 10% solutol in saline)

Olaparib-Bodipy FL imaging in a nude mouse with a HCC9137 53BP1_trunc_-Apple tumor was done as previously described^28^. Briefly, 75nmol (7.5 μl of 10 mM stock in DMSO) olaparib-Bodipy FL was diluted in 30 μl of a 1:1 dimethylacetamide (DMAC): solutol solution. PBS (112.5 μl) was then slowly added with sonication to obtain a final injection volume of 150 μl. Olaparib-Bodipy FL was injected via a tail vein catheter.

### Live cell and *in vivo* pharmacodynamic reporter analysis

Live cell imaging data and maximum intensity projections of select stacks from *in vivo* imaging were analyzed in 2D. Briefly, an in house produced MATLAB code was constructed to assist with foci counting. In this program, single cells were manually selected. Base level noise for the selected area was automatically filtered via Otsu’s method for thresholding. Foci were then automatically detected via incorporation of an automated algorithm for detecting and filtering local intensity clusters^50^, freely available from the Danuser group^51^. Results were verified by eye in each case. The average baseline number of foci prior to olaparib treatment was determined and the fold increase in foci formation (relative to day 0) was calculated for each concentration of olaparib (*in vitro*) or dose of olaparib (*in vivo*). The MATLAB code used to aid in image analysis is available upon request.

## Author Contributions

K.Y. and R.W. wrote the main manuscript text and prepared Figures 1–8 and Table 1. K.Y. and R.K. collected the data. K.Y., M.L. and R.W. analyzed and interpreted the data. R.G. and M.L. provided an automated analysis method. All authors reviewed the manuscript.

## Additional Information

**How to cite this article**: Yang, K. S. *et al*. Single cell resolution *in vivo* imaging of DNA damage following PARP inhibition. *Sci. Rep.* **5**, 10129; doi: 10.1038/srep10129 (2015).

## Supplementary Material

Supplementary Information

## Figures and Tables

**Table 1:** Relationship between BRCA status and PARP inhibitor efficacy in breast and Ewing’s sarcoma cell lines.

						**PD**_**50**_ (**μM)** ***in vitro***			
**Type**	**Model**	**BRCA1**	**BRCA2**	**PARP**	**EC**_**50**_ (**μM)** ***in vitro***	***24 hr***	***48 hr***	***6 day***	**Max fold change** ***in vivo***
Breast	MDA-MB-436	−/−	WT	Hi	0.032	0.15	0.19	0.05	3.9
	HCC1937	−/−	WT	Low	3.7	0.6	0.7	0.09	1.7
Ewing’s	MHH-ES1	WT	WT	Hi	0.16	0.02	ND	ND	2.5

Wild-type or BRCA mutant status is shown for each cell line (compiled from the COSMIC database and literature). Relative PARP expression is qualitatively compared from the Western blot in Supplementary Fig. S2. The EC_50_ value from viability assays in cell culture is also shown. The PD_50_ value (half-maximal pharmacodynamic response) was determined from imaging and quantifying the increase in foci formation following olaparib treatment and fitting the resulting curve to a dose response using GraphPad (Prism) (ND = not determined). The *in vivo* maximum fold change was determined by comparing the number of foci from pre-treatment and after daily IP injections of olaparib.
