# Supplementary material for: Single cell resolution in vivo imaging of DNA damage following PARP inhibition
Source: Sci Rep. 2015 May 18;5:10129. doi: 10.1038/srep10129 (PMC4434991; doi:10.1038/srep10129)
Supplement: Supplementary Information [file srep10129-s1.pdf]

Single cell resolution in vivo imaging of DNA damage following PARP inhibition

Katherine S. Yang, Rainer H. Kohler, Matthieu Landon, Randy Giedt, and Ralph Weissleder

Supplementary Data

Supplementary Figures

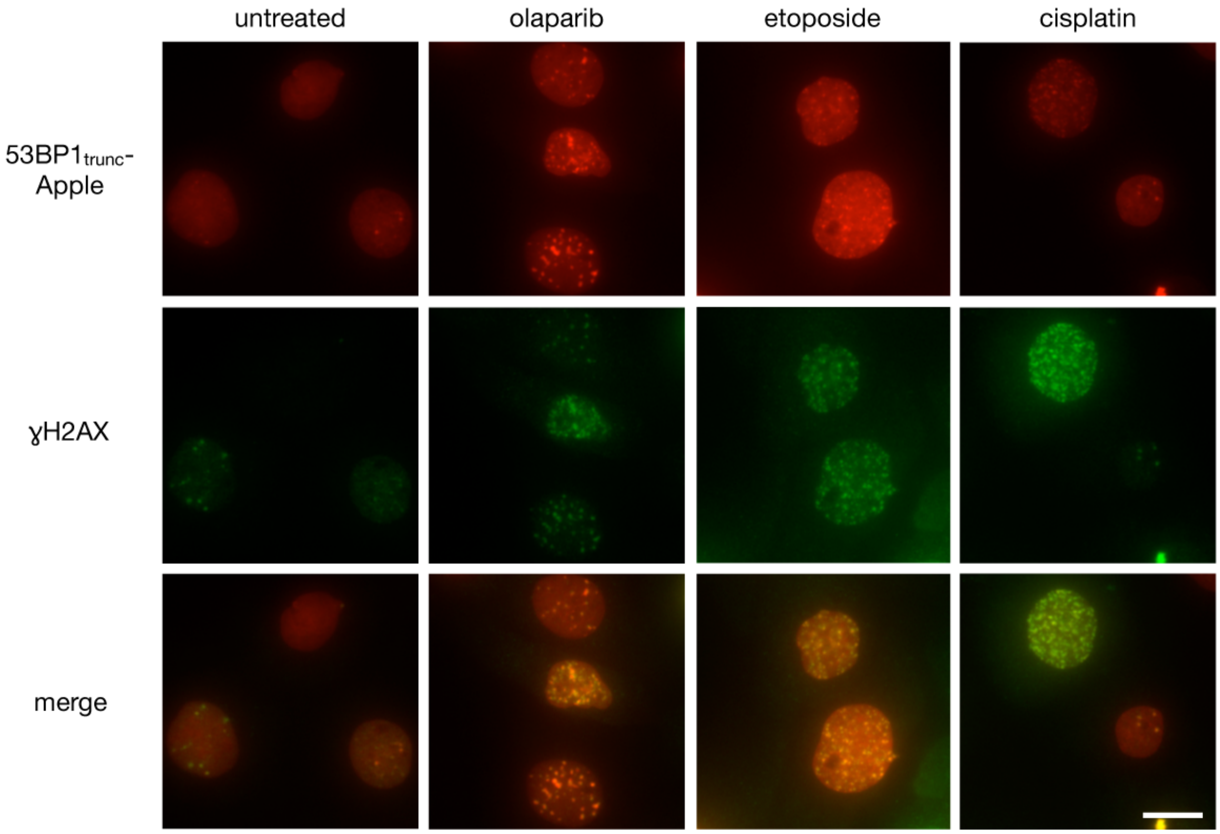

Figure S1. 53BP1$_{trunc}$-Apple reporter colocalizes with a canonical marker of DNA double-strand breaks, phospho-S139 ɣH2A.X. HCC1937 cells were treated with 0.1% DMSO (control), 100 μM olaparib or 1 μM etoposide for 24 hrs, or 10 μM cisplatin for 1 hr, followed by a 24 hr recovery. Following treatment, cells expressing 53BP1$_{trunc}$-Apple (red) were fixed and stained with an antibody against S139 phosphorylated ɣH2A.X (green, abcam, ab26350) to observe colocalization (merge) of sites of double-strand breaks. Scale bar = 20 μm.

Table S1. Panel of breast, ovarian, and Ewing's sarcoma cell lines used to examine relationship between BRCA status and PARP inhibitor efficacy. The wild-type or mutant BRCA1 and BRCA2 status is shown for each cell line (compiled from the COSMIC database and literature, ND = not determined). Relative PARP expression is compared from the Western blot in Supplementary Fig. S2. The EC_{50} value from viability assays in cell culture is also shown (Supplementary Fig. S3). The PD_{50} value (half-maximal pharmacodynamic response) was determined from imaging the increase in foci formation following olaparib treatment. The *in vivo* maximum fold change is the increase in the number of foci from daily olaparib IP injection (NG = no growth, NB = reporter not bright enough in these cell lines for in vivo use).

Type	Model	BRCA1	BRCA2	PARP	EC_{50} (μM) *in vitro*	PD_{50} (μM) *in vitro*	max fold change *in vivo*
Ovary	UWB1.289	-/-	WT	Med	0.26	0.17	NG
	A2780	WT	WT	Hi	4.7	0.03	NB
	OVCAR429	WT	WT	Low	29.4	0.63	NG
Breast	MDA-MB-436	-/-	WT	Hi	0.032	0.15	3.9
	HCC1937	-/-	WT	Low	3.7	0.6	1.7
	MDA-MB-231	WT	WT	Med	4.1	0.13	NG
Ewing's	TC-252	ND	ND	Hi	1.03	0.02	NB
	MHH-ES1	WT	WT	Hi	0.16	0.02	2.5
	SK-PN-DW	WT	WT	Low	2.4	21.7	NB

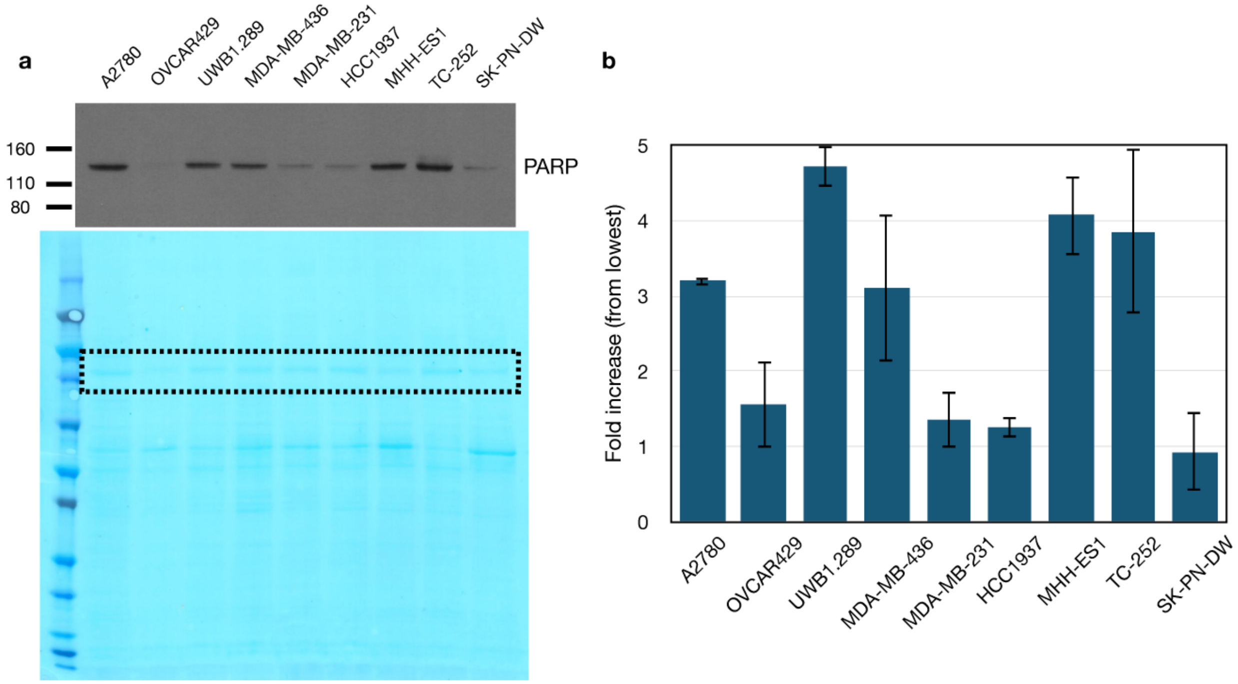

Figure S2. PARP1 protein expression in a panel of breast, ovarian, and Ewing's sarcoma cell lines. Cells were grown to confluence, lysed, and equal total protein was loaded and run on SDS-PAGE. PARP1 expression is shown for ovarian (left three lanes), breast (middle three lanes) or Ewing's sarcoma cell lines (right three lanes). PARP1 expression was quantified by densitometry using ImageJ (NIH) and normalized to a total protein membrane stain (bottom left, using the ~80kDa band outlined). Normalized PARP1 expression was plotted as a fold increase from the lowest expressing cell line (right). Error bars represent standard error of the mean from two independent experiments.

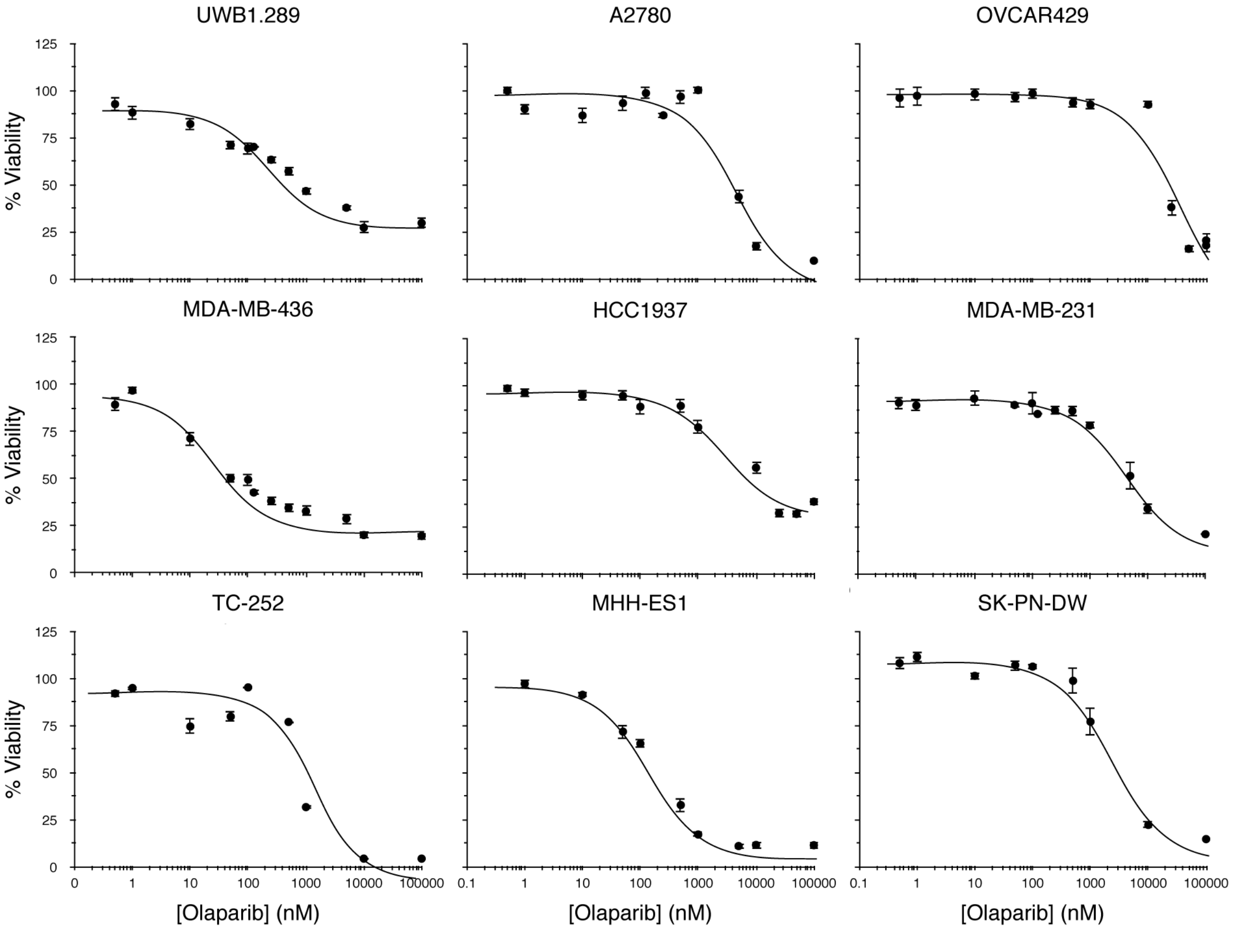

Figure S3. **Measurement of cell viability in ovarian (top row), breast (middle row) and Ewing's sarcoma cells (bottom row).** Cells were plated at low density 24 hrs prior to addition of increasing concentrations of olaparib (0 to 100 µM). After six days of treatment with olaparib, cell viability was measured using the PrestoBlue cell viability reagent (Life Technologies). The percentage of viable cells versus the concentration of olaparib was fit to a sigmoidal dose response curve using GraphPad (Prism). Error bars represent the standard error of the mean from at least two independent experiments.

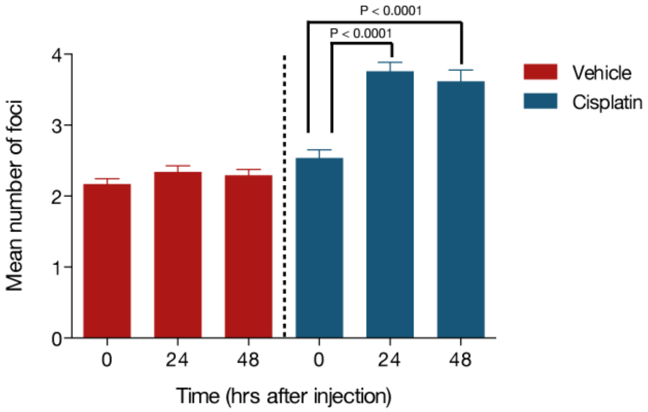

Figure S4. Validation of the 53BP1$_{trunc}$-Apple reporter *in vivo*. HT1080 cells expressing the 53BP1$_{trunc}$-Apple reporter were implanted in the window chamber of nu/nu mice and grown for approximately two weeks before imaging. Mice were either in the vehicle (red, n = 3) or cisplatin treatment (blue, n = 3) group and were imaged before treatment and 24 and 48 hrs after a single bolus dose of drug (9 mg cisplatin/kg or 10% solutol in saline). The mean number of foci per nucleus at each time point and for each group is shown. At least 100 nuclei were analyzed for each mouse on each day of imaging. For the vehicle mice, no significant difference between each time point was observed (p-value > 0.05, two-tailed t-test). For the cisplatin treated mice, a significant difference between pre-treatment and 24 and 48 hrs after dosing was observed (p-value < 0.0001, two-tailed t-test). Error bars represent the standard error of the mean.

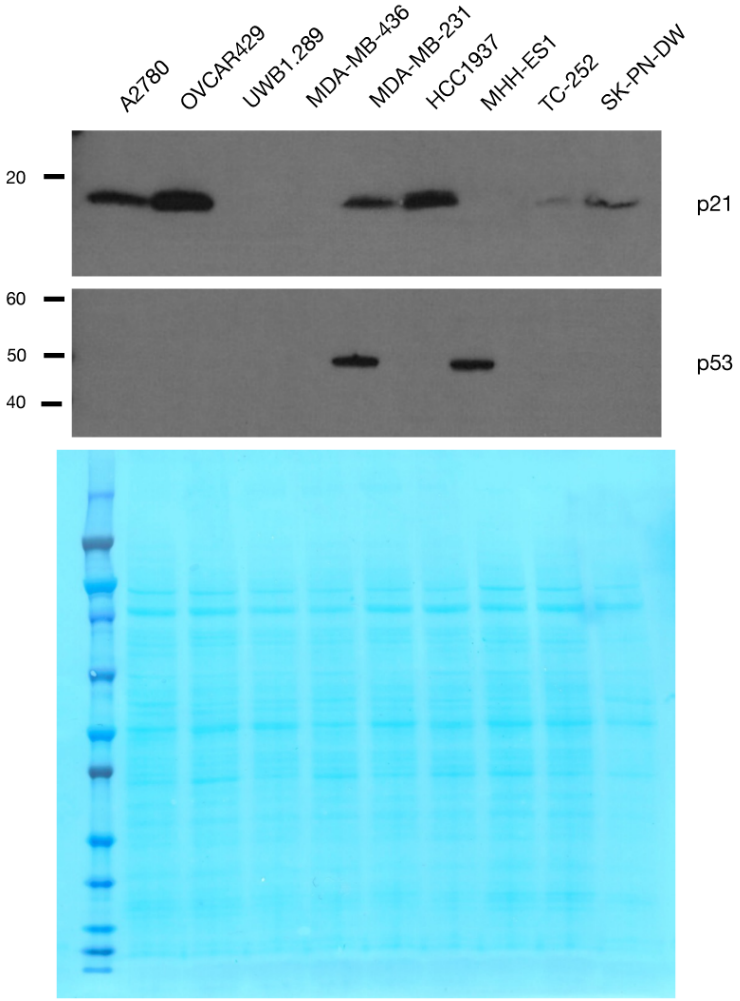

Figure S5. p21 and p53 protein expression in a panel of ovarian (left three lanes), breast (middle three lanes), and Ewing's sarcoma (right three lanes) cell lines. Cells were grown to confluence, lysed, and equal total protein was loaded and run on SDS-PAGE. Total protein membrane stain (Pierce) was used as a loading control. Blots are representative of at least two independent experiments.
